# Seasonal Variation in the Diversity of the Gut Microbiota of Short‐Faced Moles Reveals the Associations of Climatic Factors on the Gut Microbiota of Subterranean Mammals

**DOI:** 10.1002/ece3.71382

**Published:** 2025-05-07

**Authors:** Di Xu, Mengmeng Wu, Zenghao Gao, Yue Zhao, Meng Hu, Yang Wen, Linlin Wang, Deli Xu, Lei Chen

**Affiliations:** ^1^ College of Life Sciences Qufu Normal University Qufu China; ^2^ Shandong Freshwater Fisheries Research Institute Jinan China; ^3^ Forestry Protection and Development Service Center of Jining Jining China; ^4^ Jining Bureau of Natural Resources and Planning Jining China

**Keywords:** climatic factor, diversity comparison, gut microbiota, seasonal variation, short‐faced moles

## Abstract

The composition of animal gut microbiota is significantly affected by a variety of factors. Seasonal variation in environmental factors is believed to have a significant impact on the composition of mammalian gut microbiota. Therefore, studying the seasonal differences in gut microbiota diversity in wildlife is of great importance to explore their ecological adaptability. This study compared the diversity of gut microbiota of the short‐faced moles ( *Scaptochirus moschatus* ) in spring, summer, and autumn by using 16S rRNA amplification sequencing. Our results reveal significant seasonal differences in the diversity and function of the short‐faced moles gut microbiota. Compared to spring, the diversity and function of the gut microbiota in summer and autumn of short‐faced moles are more similar to each other. The relative abundance of Firmicutes is higher in spring than in summer and autumn, while the relative abundance of Proteobacteria in summer and autumn is higher than that of spring. There are significant differences in carbohydrate metabolism between spring and summer, and between spring and autumn. The correlation analysis results suggest that climatic factors are strongly associated with seasonal variation in gut microbiota of the short‐faced moles, especially temperature and relative humidity. The present study discusses the seasonal variations in the gut microbiota diversity of short‐faced moles and the significant impact of climatic factors on gut microbiota diversity. These results will highlight the potential impact of climatic factors on the seasonal changes of the gut microbiota of subterranean mammals and provide a new view for comprehensively understanding the ecological adaptation of subterranean mammals.

## Graphical abstract

Significant seasonal differences in the diversity of the gut microbiota of the short‐faced moles. Compared to spring, the diversity and function of gut microbiota in summer and autumn are more similar to each other. Climatic factors are found to have a significant association with the seasonal variation of the gut microbiota of short‐faced moles, especially temperature and relative humidity.

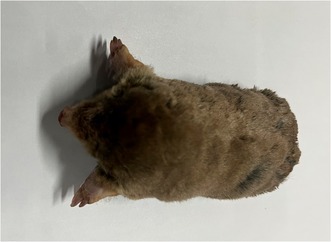

## 1 Introduction

The gastrointestinal tract is the main organ responsible for nutrient digestion and absorption in animals, and it harbors a diverse microbial community (Zhu et al. 2021; Ha et al. 2014). The gut microbiota has developed a ‘symbiotic’ relationship with its host over a long period of evolution, which can not only obtain nutrients from the host, but also help the host to digest food (Kohl et al. 2014; Funkhouser and Bordenstein 2013). The gut microbiota, known as “the second genome” of its host, plays an important role in maintaining intestinal homeostasis, combating pathogen invasion, and preventing intestinal inflammation (Kaakoush 2015; McKenney and Pamer 2015; Guan et al. 2017; Bradley and Pollard 2017). Previous studies have demonstrated that various factors, such as age (He et al. 2019), environment (Zhao et al. 2018), genetics (Grieneisen et al. 2021), and diet (Muegge et al. 2011), all influence the diversity of mammalian gut microbiota. Vertical transmission occurs primarily through the offspring's exposure to their mother's feces or vaginal microbes during delivery, resulting in a gut microbiota that is more similar to that of their mother, than to the general population (Dominguez‐Bello et al. 2010; Funkhouser and Bordenstein 2013; Smits et al. 2017). Different habitat environments result in significant geographic differences in the gut microbiota of the same specifics (Gao et al. 2020).

Seasonal variation plays a significant role in shaping the structure of the mammalian gut microbial community and is considered to be the important driving factor for changes in the gut microbial community structure (Maurice et al. 2015; Ren et al. 2017; Fan et al. 2022). Research has shown that there were significant seasonal differences in the gut microbiota of plateau pikas, with the highest diversity of gut microbiota in winter (Fan et al. 2022). The gut microbiota of the Hadza hunter‐gatherer group of Tanzania showed a cyclical pattern with the seasons, particularly Bacteroidetes (Smits et al. 2017). The gut microbiota of the wild rhesus macaques ( *Macaca mulatta* ) showed higher diversity and richness during the dry season than during the rainy season, likely due to seasonal variations in diet (Li et al. 2021). In Siberian flying squirrels ( *Pteromys volans orii* ), the order Clostridiales and the genera *Coprococcus* and *Roseburia* dominated at low temperatures, while at high temperatures, *Clostridiales* and a few families in the Erysipelotrichaceae dominated (Liu et al. 2019).

The short‐faced mole ( *Scaptochirus moschatus* ) is a small subterranean mammal of the family Talpidae, which is endemic to China. Their vision is largely degraded, but the senses of smell and hearing are relatively developed. Their unique morphological features make them an ideal subject for studying the environmental adaptations of subterranean mammals (Kawada et al. 2002). Previous studies have initially reported the gut microbiota of the short‐faced moles and found that their gut microbiota varies with the sampling sites (Chen et al. 2021). It has been reported that seasonal variation in climatic factors significantly affects the gut microbiota of small mammals (Fan et al. 2022; Ren et al. 2017). However, it is currently unclear whether seasonal variation in climatic factors can result in seasonal differences in the gut microbiota of subterranean mammals. Therefore, we collected intestinal samples from short‐faced moles during the spring, summer, and autumn and used 16S rRNA amplification sequencing to analyze the differences in gut microbial diversity and function among different seasons. Spearman correlation analysis was conducted to explore the correlation between seasonal changes in environmental factors and the difference in the diversity of short‐faced moles' gut microbiota. The results of this study will provide scientific data for the investigation of the influence of environmental factors on the diversity of the gut microbiota in subterranean mammals and promote the understanding of the ecological adaptation of subterranean mammals.

## 2 Methods

### 2.1 Sample Collection

Samples of short‐faced moles were collected from Liaocheng City, Shandong Province, China, during 2021 to 2022. All samples were divided into 3 groups: spring (SP), summer (SU), and autumn (AU), with six samples in each group (Table S1). However, samples were not collected in winter, and the specific reasons are still under further study. The short‐faced moles and their habitat were not harmed during the sampling collecting. Following the guidelines of the American Veterinary Medical Association (AVMA) guidelines for the euthanasia of animals, intraperitoneal injection of sodium pentobarbital (200 mg/kg) was used to euthanize the short‐faced moles (Zatroch et al. 2017). The entire intestinal contents of the short‐faced moles were collected for DNA extraction and sequencing. This study adheres to Chinese laws and the requirements of the China Animal Protection Association.

### 2.2 Genomic DNA Extraction and PCR Amplification

In this study, the cetyltrimethylammonium bromide (CTAB) method was employed to extract genomic DNA from the intestinal contents of short‐faced moles. Subsequently, agarose gel electrophoresis was used to determine the concentration of the extracted DNA. The DNA was transferred into a centrifuge tube and diluted with sterile water to a concentration of 1 ng/μL. Polymerase chain reaction (PCR) amplification of the 16S rRNA V3–V4 region was performed using specific primers with a barcode (515F and 806R). Then, the concentration of the PCR product was detected using 2% agarose gel electrophoresis (Figure S1). The target bands were subsequently retrieved using a gel extraction kit (Qiagen, Germany). Fragment libraries were constructed by using the NEBNext® Ultra^TM^ II DNA Library Prep Kit, and the libraries were quantified by using Qubit and Q‐PCR. Once the qualified library had been calculated, the Illumina NovaSeq sequencing platform was performed for double‐terminal sequencing.

### 2.3 ASVs Clustering, Quality Control and Species Annotation

Based on the barcode and primer sequences, each sample's data was separated, and the PE reads of each sample were spliced by using FLASH software to obtain Raw Tags (Magoc and Salzberg 2011). The Tags sequence obtained by splicing underwent quality control by using the Fastp software, resulting in the acquisition of high‐quality Clean Tags. Finally, the Vsearch software was utilized to compare the Clean Tags with the database, detect and remove chimeras, and obtain Effective Tags for subsequent analysis. The DADA2 module of the QIIME2 software was used for noise reduction (Callahan et al. 2016). Sequences with an abundance of less than five were filtered out to obtain the final Amplicon Sequence Variants (ASVs) and the ASV feature list (Li et al. 2020). The species for each ASV were annotated by using the QIIME2 classify‐sklearn algorithm (Bokulich et al. 2018). The Venn diagram visualizes the shared and unique ASVs among groups.

Based on the ASVs annotation results and the feature table, we obtained the species abundance table for each taxonomic level. Bar charts were generated by using the QIIME2 plug‐in to visualize the difference in the species annotation results among different samples at each taxonomic level. Additionally, the top 10 taxa with the highest relative abundance were selected to generate ternary plots. This allowed for a visual comparison of the dominant species among different groups (Bulgarelli et al. 2015).

### 2.4 Analysis of Gut Microbiota Diversity

QIIME2 software was utilized to calculate the alpha diversity indices (including Observed OTUs, Shannon, Simpson, Chao1, Goods coverage, Dominance, and Evenness) (Li et al. 2013), and rarefaction curves and species accumulation boxplots were plotted to evaluate the species richness and diversity of gut microbiota in different groups. Comparative analyses were conducted to determine between‐group differences in alpha diversity indices. The Tukey test was used to analyze whether there was a significant difference in the diversity of gut microbiota among groups, and box plots were plotted for visual presentation. The profiling table was created by combining ASVs of the same classification based on the species annotation results and the abundance information of the ASV feature sequences, and the weighted and unweighted unifrac distances were calculated (Lozupone and Knight 2005). Differences in beta diversity of gut microbiota among the three groups were analyzed by principal component analysis (PCA), principal co‐ordinates analysis (PCoA), and non‐metric multi‐dimensional scaling analysis (NMDS). The adonis and anosim functions of QIIME2 software were used to analyze significant differences in community structure among the three groups. To search for the biomarkers with significant differences among the three groups, the LEfSe software was utilized for LEfSe analysis in this study, with the default LDA score threshold set at 4. MetaStat analysis was performed by using R software to identify taxa with significant differences among the three groups.

### 2.5 Tax4Fun Functional Prediction

Tax4Fun is an R package that predicts the function of intestinal samples based on the 16S Silva database. A correlation matrix was established by comparing the 16S rRNA gene sequence of prokaryotes extracted from the KEGG database to the SILVA SSU Ref NR database (BLAST bitscore > 1500) by using the BLASTN algorithm. The functional information of prokaryotic organisms in the KEGG database, annotated by both UProC and PAUDA methods, was compared to the SILVA database for functional annotation. Functional annotation information of the gut microbiota of short‐faced moles was obtained by performing OTU clustering, using the SILVA database sequence as the reference sequence.

According to the annotation results, the top 10 functional annotation terms with the highest abundance at each annotation level (level 1, level 2, level 3) were selected, and the histograms of function relative abundance were plotted to visualize their relative abundance across the different annotation levels within each group. In order to analyze the common and unique information of gene function between different groups, Venn graphs were drawn for visualization. PCA analysis was used to test the analogous functional profiles of gut microbiota function between different groups, and the *T*‐test was used to determine whether there were significant differences in function between different groups.

### 2.6 Correlation Analysis of the Gut Microbiota and the Climate Data

To analyze factors that contribute to seasonal differences in gut microbiota diversity, Spearman correlation analysis was conducted between differential gut microbiota and climate data by using IBM SPSS Statistics 27. Redundancy analysis (RDA) (Yang et al. 2018; Liu et al. 2020) was also performed through the cloud platform of Shanghai Biozeron Biotechnology Co. Ltd., with a view to achieving a more comprehensive analysis of the correlation between climatic factors and gut microbiota. Climate data were sourced from the meteorological station in Liaocheng City, available from http://rp5.ru/archive.php?wmo_id=54808&lang=cn. The climate data utilized in the correlation analysis are daily averages from the day on which the samples were collected (Table S2).

## 3 Results

### 3.1 Species Annotation

In this study, a total of 1,460,698 raw tags were obtained. After splicing and filtering out low‐quality and short‐length sequences, 1,356,529 clean tags were obtained. A total of 1,221,566 effective tags were obtained for subsequent analysis.

**FIGURE 1:**
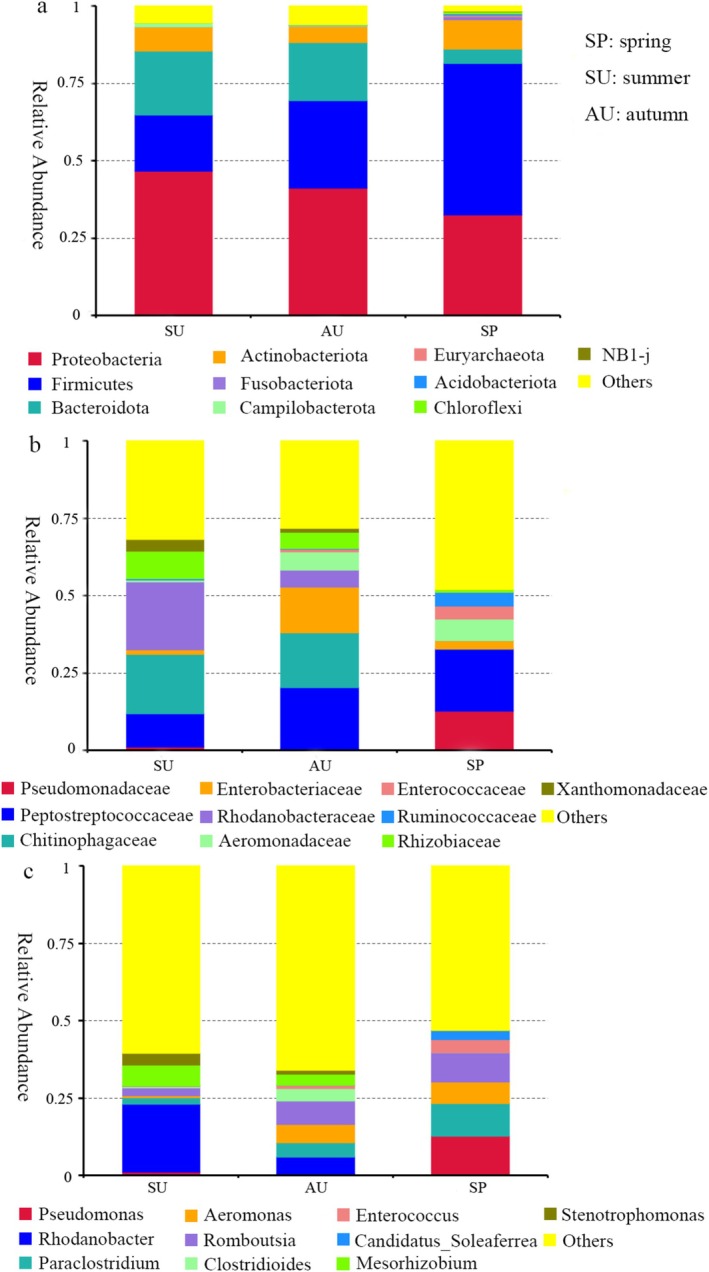
The composition of gut microbiota at the phylum (a), family (b), and genus (c) levels in each group.

A total of 35 phyla, 226 orders, 370 families, 720 genera, and 314 species have been annotated in the gut microbiota of the short‐faced moles. The dominant phyla are Proteobacteria, Firmicutes, Bacteroidota, Actinobacteriota, Fusobacteriota, and Campilobacterota. The relative abundance of Proteobacteria, Bacteroidota, and Campilobacterota in the gut microbiota of the short‐faced moles in SP is lower than that in SU and AU, while the relative abundance of other phyla is higher than that in SU and AU. Euryarchaeota and NB1‐j are present only in the group SP and are not found in the groups SU and AU (Figure 1a). At the family level, the relative abundance of Pseudomonadaceae, Peptostreptococcaceae, Aeromonadaceae, Enterococcaceae, and Ruminococcaceae in the gut microbiota of short‐faced moles in SP is higher than that in SU and AU. The relative abundance of Enterobacteriaceae in the AU group is higher than that in SP and SU. The relative abundance of Rhodanobacteraceae, Chitinophagaceae, Xanthomonadaceae, and Rhizobiaceae is higher in the SU group than that in SP and AU (Figure 1b). At the genus level, the relative abundance of *Stenotrophomonas*, *Mesorhizobium*, *Clostridioides*, and *Rhodanobacter* is lower in SP than that in SU and AU. Additionally, the relative abundance of *Rhodanobacter*, *Stenotrophomonas*, and *Mesorhizobium* is higher in SU than in SP and AU. Conversely, genus *Clostridioides* has a higher relative abundance in the AU group than in SP and SU (Figure 1c). A total of 162 ASVs are shared in three seasons, including the genera *Akkermansia*, *Parabacteroides*, *Bacteroides*, *Blautia*, *Faecalibacterium*, *Dubosiella, Romboutsia, Allobaculum, Lactobacillus*, and others (Figure S2).

### 3.2 Analysis of Gut Microbiota Diversity

The alpha diversity indicates that the Chao 1, Observed OTUs, Evenness, and Shannon indices are higher in SP than in SU and AU, which suggests that the diversity of the gut microbiota of the short‐faced moles is higher in spring (Table 1).

**TABLE 1:** The alpha diversity index of each group.

Group	Chao1	Dominance	Goods coverage	Observed OTUs	Evenness	Shannon	Simpson
SP	627.02	0.14	1	624.67	0.60	5.53	0.86
SU	367.10	0.14	1	363.83	0.53	4.46	0.86
AU	325.64	0.11	1	323.17	0.58	4.82	0.89

The Tukey test of alpha diversity indices and beta diversity analysis were used to compare the difference of gut microbiota among different groups. The Tukey tests indicate that there are significant differences in the Chao 1 index and the Observed OTUs of gut microbiota between the SP and SU, as well as the SP and AU (*p* < 0.05), but no significant difference in Shannon, Simpson, Goods coverage, Dominance, and Evenness indices among the three groups (Figure S3).

**FIGURE 2:**
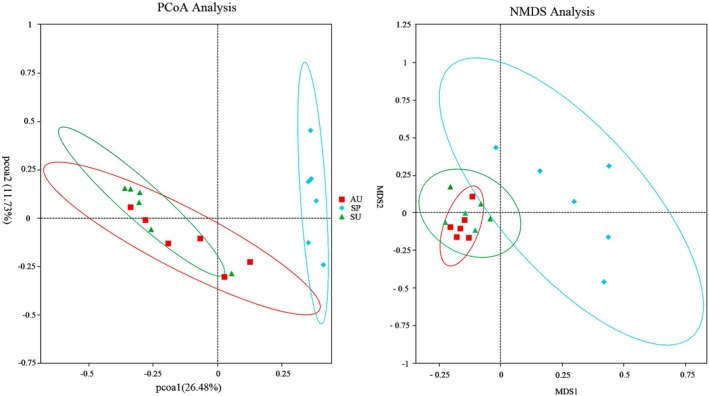
PCoA analysis and NMDS analysis based on the Bray‐Curtis distance.

The PCoA and NMDS analyses, based on the Bray‐Curtis distance, indicate that the samples from the SP group are clustered together, while samples from the SU and AU groups cluster together (Figure 2). Both UPGMA cluster trees based on weighted unifrac and unweighted unifrac distances all show significant differences in the gut microbiota of short‐faced moles between spring and summer, and between spring and autumn (Figure S4).

### 3.3 Analysis of Species Differences Among Groups

The MetaStat hypothesis test was performed to analyze which species contribute significantly to seasonal differences in gut microbiota. The results indicate that there is more microbiota that have significant differences between spring and summer, or between spring and autumn than that between summer and autumn.

At the phylum level, significant differences are observed between SP and AU in the abundance of Bacteroidota, Chloroflexi, Cyanobacteria, Gemmatimonadota, and Deinococcota. The relative abundance of Bacteroidota and Deinococcota is higher in AU than in SP, while the relative abundance of Chloroflexi, Cyanobacteria, and Gemmatimonadota is higher in SP than in AU. The relative abundance of Bacteroidota and Verrucomicrobiota in group SU is higher than that in SP. The relative abundance of Firmicutes, Acidobacteriota, Chloroflexi, and Gemmatimonadota in group SP is higher than that in group SU. Only phylum Desulfobacterota showed a significantly difference between group SU and group AU, and its relative abundance is higher in group SU than that in group AU (Figure S5).

At the family level, there are 52 families with significant differences between SP and AU, including Chitinophagaceae, Rhodanobacteraceae, Ruminococcaceae, Rhizobiaceae, Xanthomonadaceae, Lachnospiraceae, etc. Significant differences are found between SP and SU in 56 families, such as Micrococcaceae, Caulobacteraceae, Bacillaceae, Methyloligellaceae, etc. There are significant differences between SU and AU in Rhodanobacteraceae, Muribaculaceae, Flavobacteriaceae, Alcaligenaceae, Gitt‐GS‐136, and TK10 (Table S3).

**FIGURE 3:**
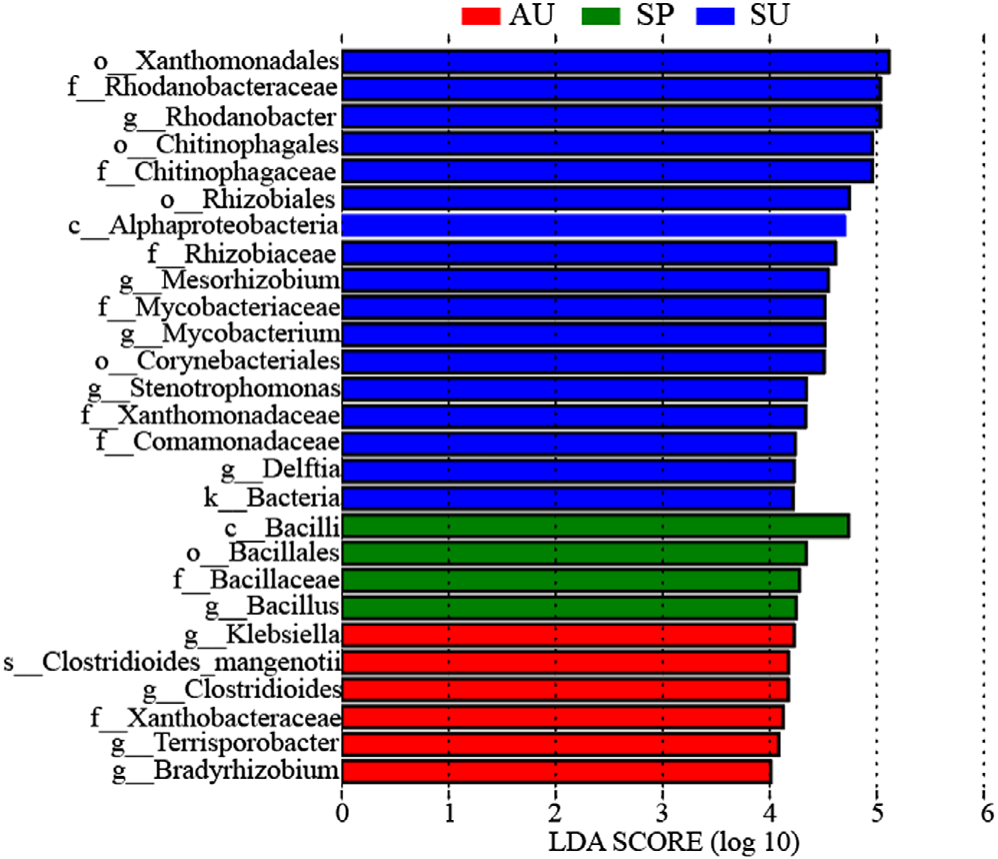
LEfSe analysis among SP, SU and AU groups.

The biomarkers with statistically significant differences in the gut microbiota between groups are Actinobacteriota, Bacteroidota, Firmicutes, and Proteobacteria. The main biomarkers are orders Xanthomonadales, Chitinophagales, and Rhizobiales, families Rhodanobacteraceae, Chitinophagaceae, Rhizobiaceae, and Mycobacteriaceae, and genera *Rhodanobacter*, *Mesorhizobium*, and *Mycobacterium* in the SU group, class Bacilli, family Bacillaceae, and genus *Bacillus* in the SP group, the family Xanthobacteraceae, and the genera *Klebsiella*, *Clostridioides*, and *Terrisporobacter* in the AU group (Figure 3).

### 3.4 Correlation Analysis of the Gut Microbiota and the Climatic Factors

**FIGURE 4:**
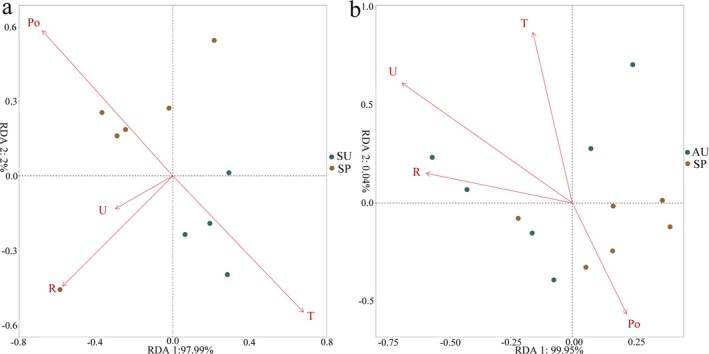
The RDA analysis between the SP and SU groups (a), as well as the SP and AU groups (b).

To analyze the main factors causing seasonal differences in the gut microbiota of short‐faced moles, Spearman correlation analysis was performed to examine the correlation between the gut microbiota and the climatic factors. Among the gut microbiota with significant differences between SP and SU, Acidobacteriota, Chloroflexi, and Patescibacteria are found to be significantly correlated with temperature (T) and atmospheric pressure (Po). The phylum Acidobacteriota and Verrucomicrobiota have a significant negative correlation with the relative humidity (U), and the phylum Bacteroidota has a significant negative correlation with the atmospheric pressure. The temperature and atmospheric pressure have opposite effects on gut microbiota. Among the gut microbiota with significant differences between SP and AU, Cyanobacteria show a significant negative correlation with relative humidity and rainfall (R). Chloroflexi and Gemmatimonadota have a significant negative correlation with the relative humidity. The Desulfobacterota is significantly different between SU and AU, which is related to the influence of the relative humidity of the environment (Table S4). The results of the RDA analyses also indicate that temperature, relative humidity, and atmospheric pressure are significant factors influencing the differences in gut microbiota between spring and summer. Relative humidity is a crucial factor affecting the differences in gut microbiota between spring and autumn seasons (Figure 4).

### 3.5 Functional Diversity Analysis of Gut Microbiota

Tax4Fun was utilized to predict the function of the gut microbiota of short‐faced moles. A total of 6547 functional pathways are shared in the SP, SU, and AU groups, including the fatty aldehyde‐generating acyl‐ACP reductase, transcriptional regulator of arginine metabolism, NADPH2: quinone reductase, and other related pathways. A total of 47 functional pathways are only observed in the SP group, including energy‐converting hydrogenase A subunit B, hypothetical protein, bacteriorhodopsin, and others. The creatine kinase and 2‐hydroxycinnamic acid beta‐D‐glucosylisomelase functional pathways are shared between the SU and AU groups (Figure S6).

**FIGURE 5:**
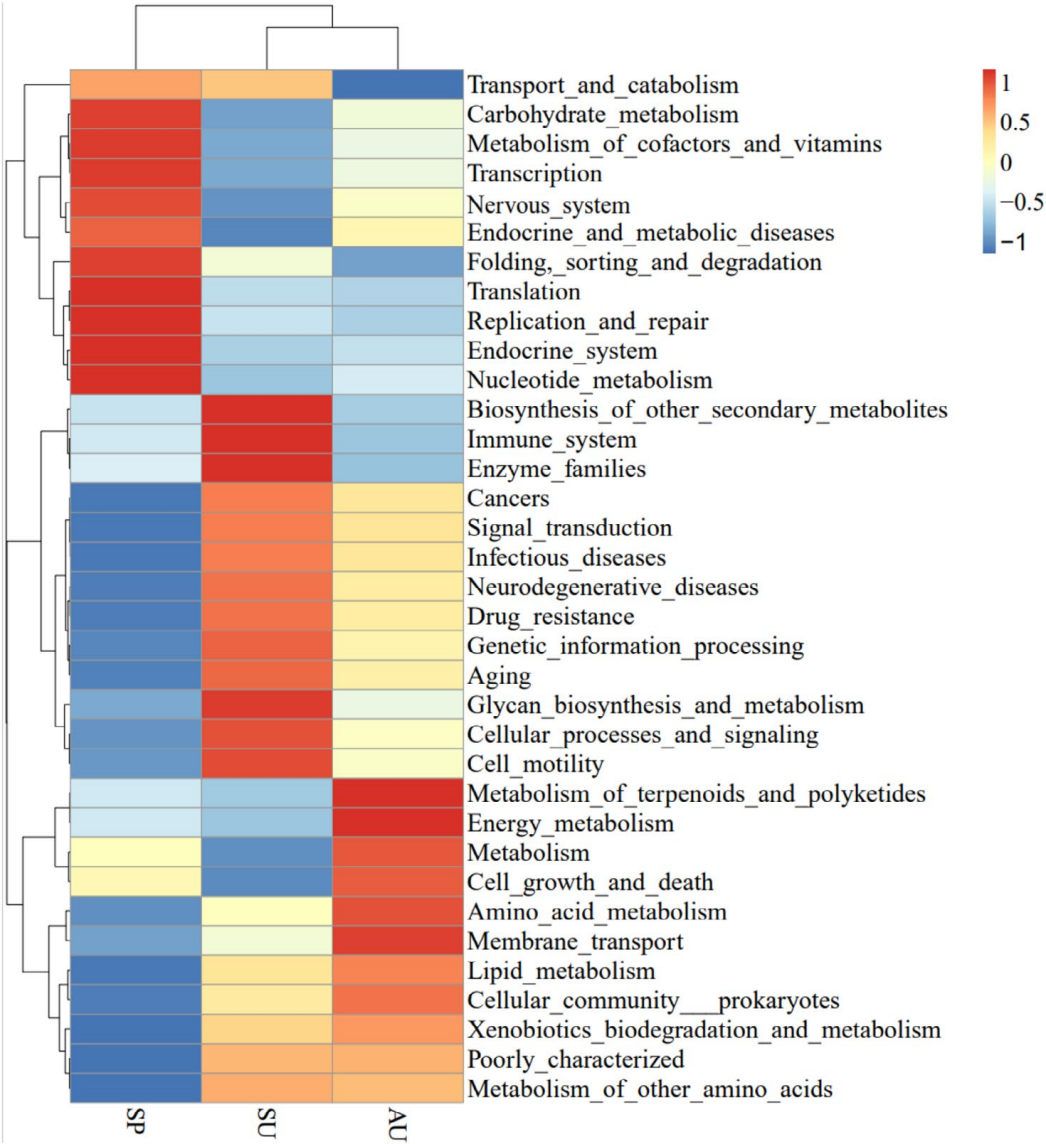
Functional annotation information of the gut microbiota.

**FIGURE 6:**
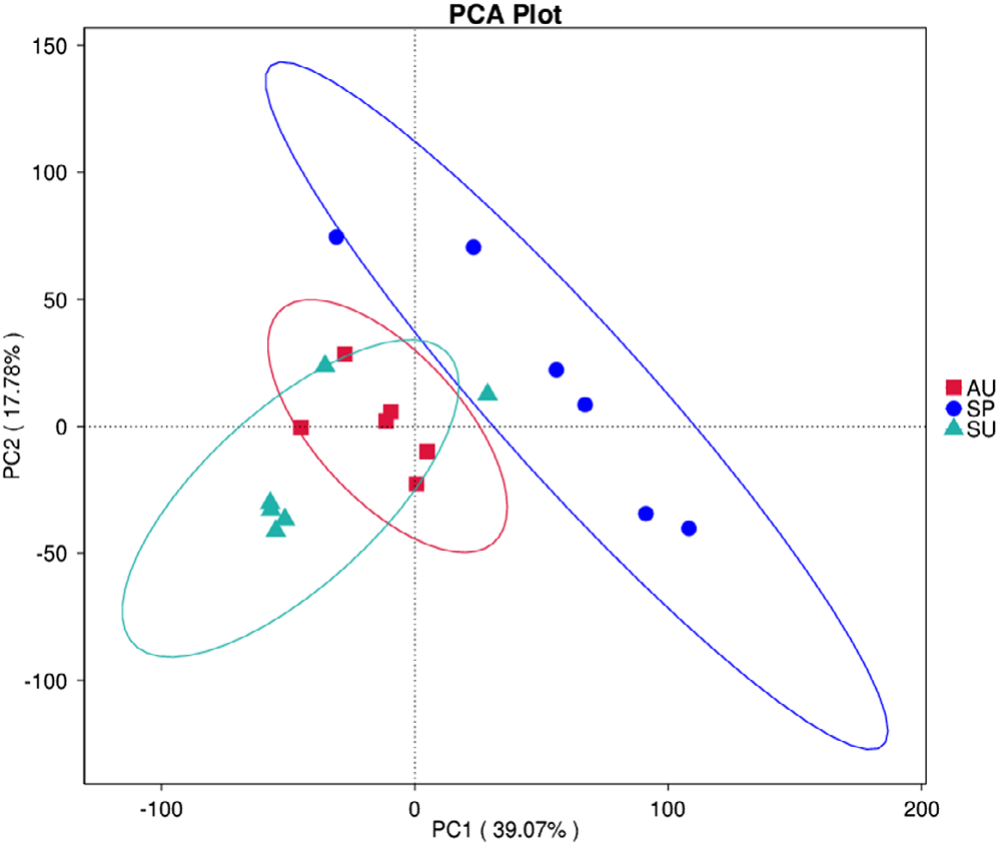
PCA analysis of functional information.

**FIGURE 7:**
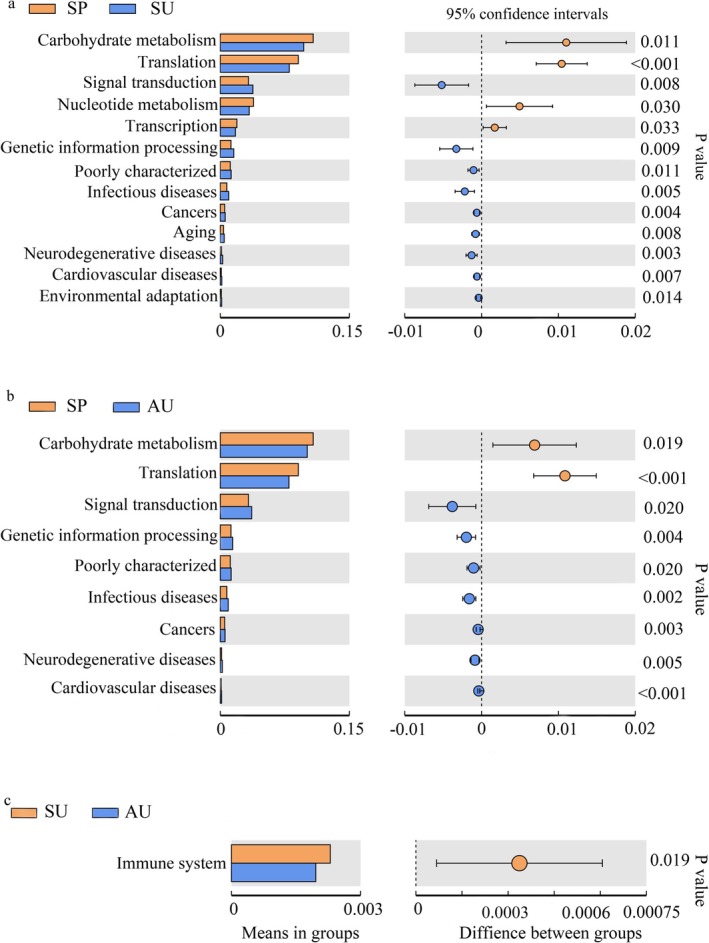
*T*‐test of functional information (a. SP vs. SU, b. SP vs. AU, c. SU vs. AU).

The relative abundance of functional pathways, including carbohydrate metabolism, metabolism of cofactors and vitamins, translation, replication and repair, and nucleotide metabolism, is higher in the SP group than in SU and AU. Compared to the SP and AU groups, the relative abundance of biosynthesis of other secondary metabolites, immune system, glycan biosynthesis and metabolism, cellular processes and signaling functional pathways is higher in the SU group. The relative abundance of metabolism of terpenoids and polyketides, energy metabolism, cell growth and death, amino acid metabolism, and lipid metabolism pathways is higher in the AU group than in the SP and SU groups (Figure 5). PCA results show that the functions of the gut microbiota are more similar between SU and AU than those between SP and SU, and between SU and AU, which is consistent with the PCA results of the diversity analysis of gut microbiota (Figure 6). The functional pathways of carbohydrate metabolism, translation, signal transduction, and genetic information processing are found to be significantly different between the SP and SU groups and between the SP and AU groups by t‐test (*p* < 0.05). The only functional pathway that shows a significant difference between groups SU and AU is the immune system (Figure 7).

## 4 Discussion

In this study, our results show that the dominant phyla in the gut microbiota of short‐faced moles are Proteobacteria, Firmicutes, Bacteroidota, and Actinobacteriota, which is consistent with the results of our previous study on the gut microbiota of short‐faced moles (Chen et al. 2021). Firmicutes and Proteobacteria are found to comprise over 64% of the gut microbiota of short‐faced moles, particularly in the spring (with a proportion of 81.5%). These two phyla were also dominant in the gut microbiota of other wild mammals, such as plateau pikas ( *Ochotona curzoniae* ) (Ren et al. 2024), naked mole‐rat ( *Heterocephalus glaber* ) (Debebe et al. 2017), and blind mole rat (Kuang et al. 2022). Previous research indicated that Proteobacteria is closely associated with food high in sugar and fat (Wu et al. 2022; Filippo et al. 2010; Han et al. 2019; Bulgarelli et al. 2015). In the present study, Proteobacteria is more abundant during the summer and autumn. The results of Tax4Fun functional analyses also indicate that the functional pathways related to lipid metabolism are significantly higher in summer than in spring. Therefore, we hypothesize that the high abundance of Proteobacteria might be related to the high‐fat food in summer.

The Firmicutes is associated with protein and cellulose degradation, as well as amino acid and carbohydrate metabolism (Trujillo et al. 2022; Sugden et al. 2021; Tanes et al. 2021). Bacteroidota is responsible for the digestion and breakdown of carbohydrates, which is closely linked to high‐fat, high‐sugar, and other high‐calorie diets (Guan et al. 2017; Ni et al. 2020; Ley et al. 2006). Previous studies have shown that members in the phyla Firmicutes and Bacteroidota may contribute to more efficient energy absorption and transformation. The ratio of Firmicutes to Bacteroidota (F/B) is often considered a significant indicator of animal obesity due to its association with the accumulation of animal fat (Ley et al. 2006; Gao et al. 2020; Wolf et al. 2021; Riva et al. 2017; Chen et al. 2016). In this study, we find that the value of F/B in summer (0.88) is lower than that in autumn (1.52) and spring (10.62), which is similar to that of the plateau pikas (Ren et al. 2024). The higher F/B ratios were observed in the obese Damaraland mole‐rats (*Fukomys damarensis*) (Bensch et al. 2023). Thus, the fat content of the short‐faced moles may be lower in summer than in spring and autumn. We speculate that short‐faced moles may begin to store fat in the autumn to cope with the cold winter environment and prepare for breeding in the coming year.

The present study reveals that there are significant seasonal variations in the diversity of the short‐faced moles' gut microbiota, and its diversity is found to be more similar in summer and autumn. The *Bacillus* genus is identified as a biomarker in spring. A number of species within the *Bacillus* genus have been observed to exhibit a range of hydrolytic and fermentative capabilities (Krishnamurthi and Chakrabarti 2013). *Bacillus velezensis* is known to produce a variety of enzymes, antibiotics, and other metabolites, and has been demonstrated to play an important role in host health (Bensch et al. 2023). The *Bacillus aerolatus* CX253 has been demonstrated to enhance the relative abundance of probiotics (such as *Lactobacillus*, etc.), and the levels of short‐chain fatty acids, thereby maintaining gut microbiota homeostasis (Yu et al. 2024). Moreover, *Bacillus subtilis* SWL‐19 is capable of producing a range of digestive enzymes such as protease, amylase, and others that facilitate the breakdown and digestion of host food (Li et al. 2023). The functional prediction results show that functional pathways related to carbohydrate metabolism are higher in spring than in summer and autumn. Therefore, we hypothesize that the high abundance of *Bacillus* in spring may promote carbohydrate metabolism.

Previous studies found that the seasonal change in the mammalian gut microbiota was mainly influenced by the seasonal alterations in food (Baniel et al. 2021; Fan et al. 2022; Ren et al. 2017; Smits et al. 2017; Liu et al. 2019). The gut microbiota diversity of woodrats (*Neotoma* spp.) changed with the seasonal variation in their diet. Because there were more secondary metabolites in the food of woodrats during the dry season, the diversity of gut microbiota was higher in the dry season than in the rainy season (Klur and Dearing 2023). The gut microbiota of plateau pikas also showed significant seasonal differences, which may be caused by seasonal changes in diet and altitude (Ren et al. 2024). However, the impact of seasonal variation in climatic factors, such as temperature and rainfall, on gut microbial diversity still requires much attention (Grieneisen et al. 2021; Orkin et al. 2019). Climate warming has been found to negatively impact the diversity of gut microbiota in ectotherms. Specifically, the abundance of gut microbiota can be significantly decreased by approximately 34% with a temperature increase of 2°C–3°C (Bestion et al. 2017). Seasonal variations in the gut microbiota of Siberian flying squirrels ( *Pteromys volans orii* ) were significantly correlated with changes in seasonal temperature, and most of the gut microbiota that are associated with temperature changes belonged to the Firmicutes (Liu et al. 2019). In addition, the gut microbiota of gelada ( *Theropithecus gelada* ) responded rapidly to seasonal climate changes, especially rainfall, that resulted in approximately 3.3% alteration of the gut microbiota (Baniel et al. 2021). And 8.93% of the variation in the gut microbiota of wild François' langurs ( *Trachypithecus francoisi* ) could be explained by rainfall and humidity, which were found to be the most significant climatic factors affecting the langurs' gut microbiota (Liu et al. 2023). Our results also indicate a significant correlation between the climatic factors (particularly temperature and relative humidity) and the diversity of gut microbiota. The diversity of gut microbiota is mainly affected by relative humidity and rainfall between spring and autumn, especially for Cyanobacteria, which is significantly negatively correlated with relative humidity and rainfall. The differences in gut microbiota diversity of the short‐faced moles between spring and summer are associated with temperature and atmospheric pressure, with relative humidity exhibiting a comparatively minor effect. Acidobacteriota, Chloroflexi, and Patescibacteria have a significant positive correlation with temperature between spring and summer. Acidobacteriota also shows a significant negative correlation with relative humidity. Firmicutes exhibit a negative correlation with temperature and a positive correlation with relative humidity, although the correlation is not statistically significant. It is found that, as the temperature increases, the relative abundance of Firmicutes decreases and the relative abundance of Bacteroidota increases. The apparent effects of temperature on Firmicutes and Bacteroidota are consistent with that of most vertebrates (Dietz et al. 2022; Al‐khlifeh et al. 2023; Fang et al. 2023). The effects of temperature on the gut microbiota of wildlife may be influenced by host metabolism, which in turn affects the host's energy utilization (Dietz et al. 2022). The ratio of Firmicutes to Bacteroidota increases with decreasing temperature, which can lead to higher energy consumption and facilitate their adaptation to cold environments (Dietz et al. 2022; Chevalier et al. 2015).

In addition, our results show that atmospheric pressure plays a very important role in causing differences in gut microbiota between spring and summer. There is a significant negative correlation between Bacteroidota and atmospheric pressure. Anatolian blind mole rats (*Nannospalax xanthodont*) showed a higher abundance of Bacteroidota and a lower abundance of Firmicutes at high altitude and low atmospheric pressure, which is consistent with our findings (Solak et al. 2024). Han et al. found that Firmicutes and Bacteroidota were also altered by hypoxic environments (Han et al. 2022). Under the hypoxic environments, the relative abundance of Bacteroidota increased while that of Firmicutes decreased (Wang et al. 2022). In particular, *Parabacteroides* and *Bacteroides* are significantly enriched in hypoxic environments and are sensitive to changes in oxygen concentration (Han et al. 2022, 2020; Wang et al. 2022). Our results show that *Parabacteroides* and *Bacteroides* are present in all three seasons, and there is no significant difference among them. Therefore, we hypothesize that this may also be the key to the short‐faced moles adaptation to hypoxic environments.

## 5 Conclusion

In this study, we analyzed the diversity and function of the gut microbiota of short‐faced moles among different seasons by using 16s rRNA amplicon sequencing. The results indicate significant seasonal differences in the diversity of the gut microbiota of the short‐faced moles. Compared to spring, the diversity and function of gut microbiota in summer and autumn are more similar to each other. Climatic factors are found to have a significant association on the seasonal variation of the gut microbiota of short‐faced moles, especially temperature and relative humidity. Temperature is the main factor contributing to the differences in gut microbial diversity between the spring and summer seasons, while relative humidity is the main factor contributing to the differences in spring and autumn. In addition, our results show that atmospheric pressure also plays an important role in the difference of gut microbiota between spring and summer. For subterranean species, the influence of environmental factors in their burrow is also very important. However, due to the complexity of their burrow environment, the climate data of the burrow environment cannot be monitored for a long period of time. Therefore, the surface climate data are used to indirectly reflect the climate condition of the burrow. After all, the burrow environment is affected by the surface climate (Tang et al. 2022; Belouz and Zereg 2023). The burrow structure and its internal environment of short‐faced moles are our subsequent research plan. Notwithstanding, we found that environmental factors are indeed associated with the gut microbiota of short‐faced moles. These findings presented in this paper are valuable for exploring the ecological adaptation mechanisms of subterranean mammals. These findings will contribute to the analysis of the ecological adaptation of subterranean mammals, and be valuable for exploring the seasonal variation of the subterranean mammals' gut microbiota.

## Author Contributions

**Di Xu:** data curation (equal), writing – original draft (equal), writing – review and editing (equal). **Mengmeng Wu:** methodology (equal), writing – review and editing (equal). **Zenghao Gao:** writing – review and editing (equal). **Yue Zhao:** writing – review and editing (equal). **Meng Hu:** investigation (equal). **Yang Wen:** investigation (equal). **Linlin Wang:** investigation (equal). **Deli Xu:** investigation (equal). **Lei Chen:** funding acquisition (equal), investigation (equal), project administration (equal), writing – review and editing (equal).

## Ethics Statement

This study was approved by the Bioethics Committee of Qufu Normal University (protocol code: 2024117) and adhered to Chinese laws and the requirements of the China Animal Protection Association.

## Conflicts of Interest

The authors declare no conflicts of interest.

## Supporting information


**Figure S1.** The gels image of PCR amplification products for 18 samples. The sample numbers range from 1 to 18, with repeated numbers indicating the absence of a target band in that electrophoresis. The experiment was reiterated until the target band was observed, and the identical numbering denotes the same sample. (a–e) indicates that five electrophoresis experiments were performed.
**Figure S2.** The Venn maps of gut microbiota among SP, SU and AU groups.
**Figure S3.** The box plots of Chao 1 index (a) and Observed OTUs index (b).
**Figure S4.** The UPGMA cluster trees based on weighted unifrac and unweighted unifrac distance.
**Figure S5.** MetaStat analysis of different groups at the phylum level.
**Figure S6.** The Venn maps of functional pathways among SP, SU and AU groups.

Tables S1–S4.

## Data Availability

The genome sequence data of this study can be obtained from the GenBank of NCBI (https://www.ncbi.nlm.nih.gov/) under the accession number PRJNA1147398. All data analyzed during this study are included in this published article and its Supporting Information files.
