# Supplementary material for: Seasonal Variation in the Diversity of the Gut Microbiota of Short‐Faced Moles Reveals the Associations of Climatic Factors on the Gut Microbiota of Subterranean Mammals
Source: Ecol Evol. 2025 May 7;15(5):e71382. doi: 10.1002/ece3.71382 (PMC12058457; doi:10.1002/ece3.71382)

**Supplementary figure**

Figure S1. The gels image of PCR amplification products for 18 samples. The sample numbers range from 1 to 18, with repeated numbers indicating the absence of a target band in that electrophoresis. The experiment was reiterated until the target band was observed, and the identical numbering denotes the same sample. Figures S1(a-e) indicates that five electrophoresis experiments were performed.


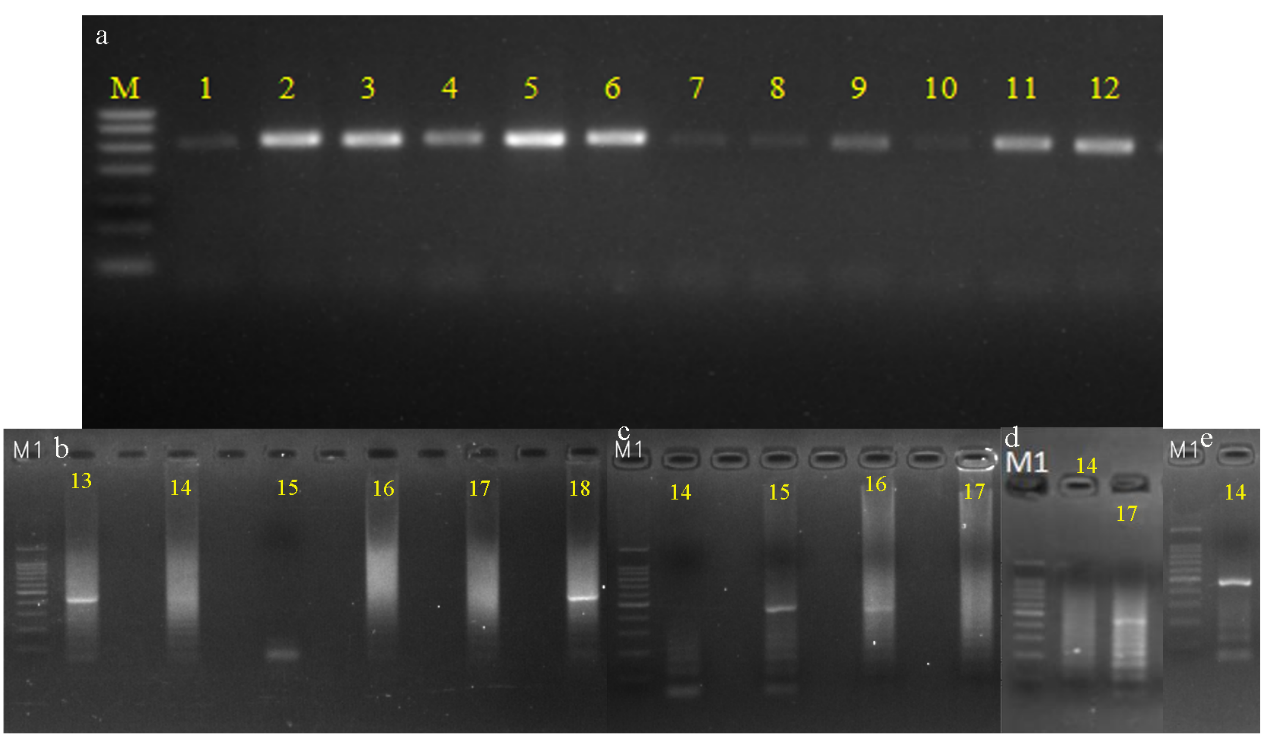


Figure S2. The Venn maps of gut microbiota among SP, SU and AU groups.


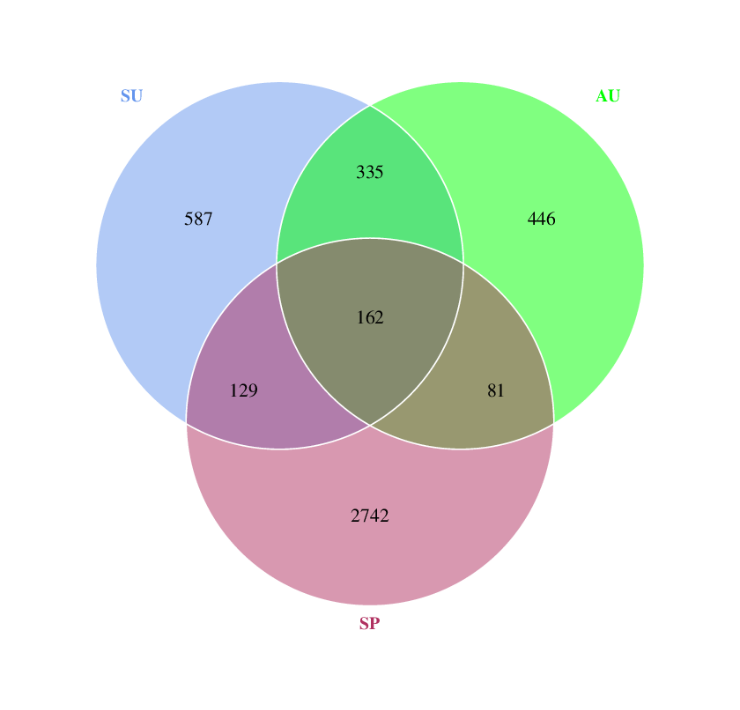


Figure S3. The box plots of Chao 1 index (a) and Observed OTUs index (b).


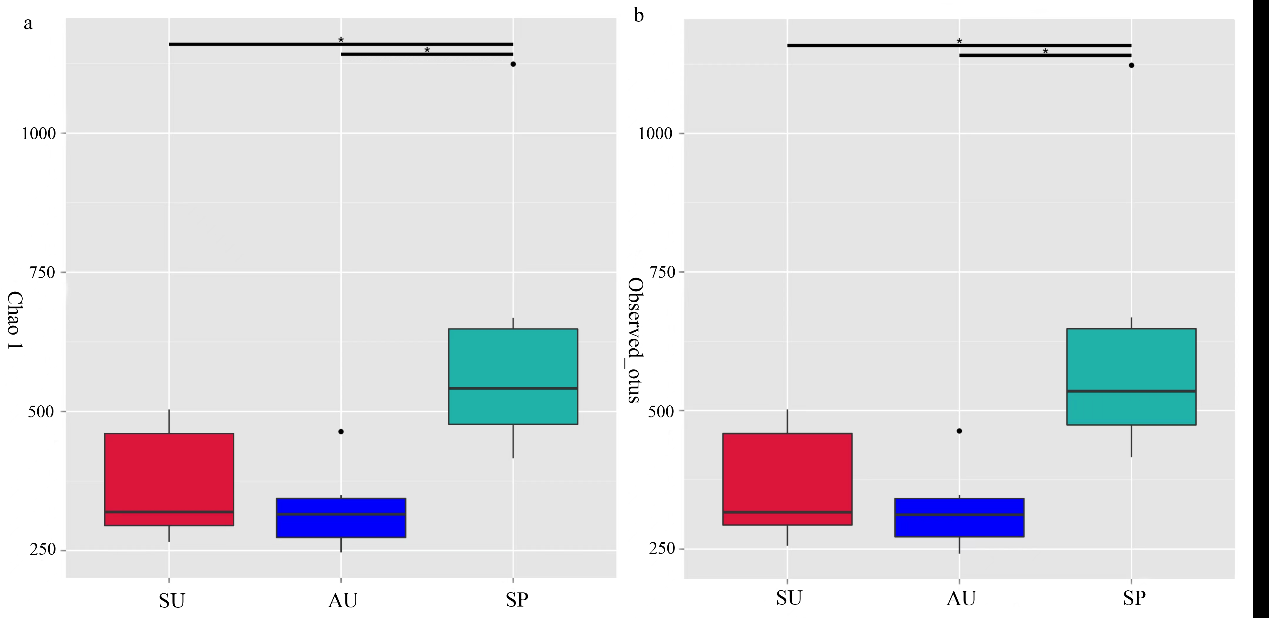


Figure S4. The UPGMA cluster trees based on weighted unifrac and unweighted unifrac distance.


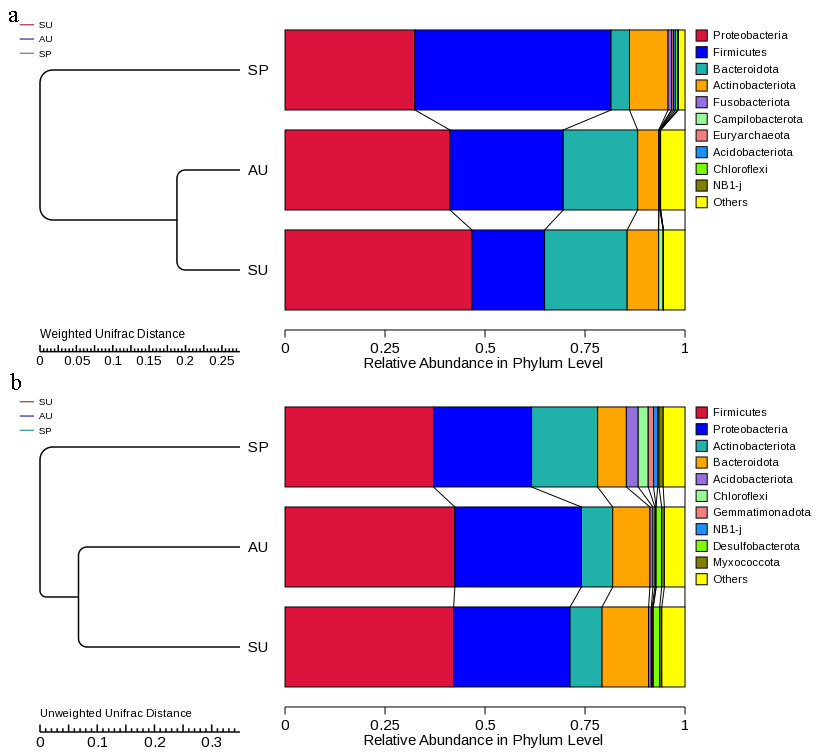


Figure S5. MetaStat analysis of different groups at the phylum level.


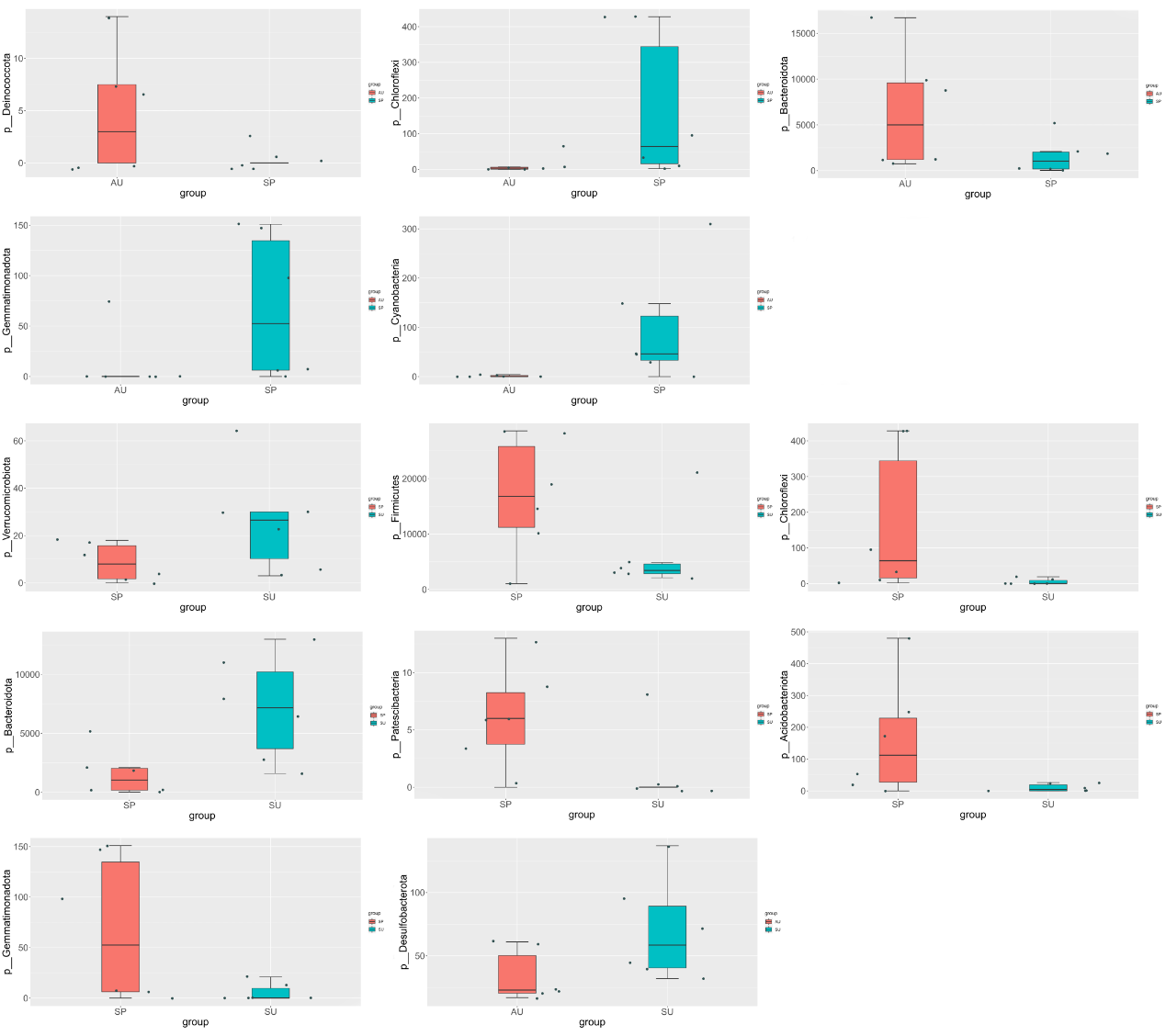


Figure S6. The Venn maps of functional pathways among SP, SU and AU groups.


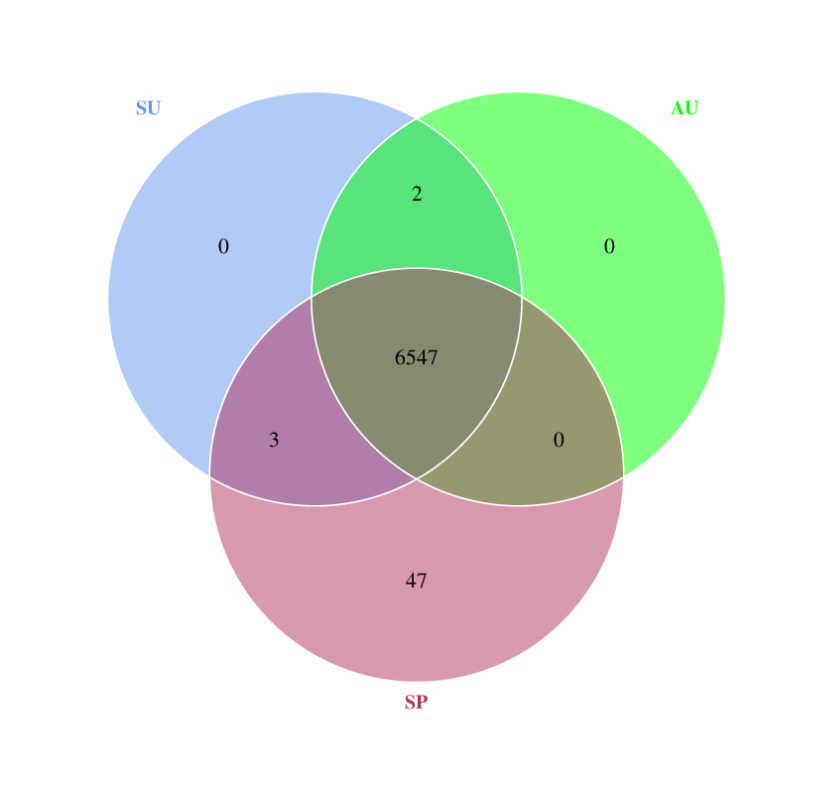

Supplement: Supplementary file 1 — Figure S1. The gels image of PCR amplification products for 18 samples. The sample numbers range from 1 to 18, with repeated numbers indicating the absence of a target band in that electrophoresis. The experiment was reiterated until the target band was observed, and the identical numbering denotes the same sample. (a–e) indicates that five electrophoresis experiments were performed. Figure S2. The Venn maps of gut microbiota among SP, SU and AU groups. Figure S3. The box plots of Chao 1 index (a) and Observed OTUs index (b). Figure S4. The UPGMA cluster trees based on weighted unifrac and unweighted unifrac distance. Figure S5. MetaStat analysis of different groups at the phylum level. Figure S6. The Venn maps of functional pathways among SP, SU and AU groups. [file ECE3-15-e71382-s002.docx]
