# Supplementary material for: Seasonal Variation in the Diversity of the Gut Microbiota of Short‐Faced Moles Reveals the Associations of Climatic Factors on the Gut Microbiota of Subterranean Mammals
Source: Ecol Evol. 2025 May 7;15(5):e71382. doi: 10.1002/ece3.71382 (PMC12058457; doi:10.1002/ece3.71382)
Supplement: Supplementary file 2 — Tables S1–S4. [file ECE3-15-e71382-s001.pdf]

Table S1. Sample information of the short-faced moles (*Scaptochirus moschatus*)

sample name	group name	sample name	group name	sample name	group name
SMSU01	SU	SMAU07	AU	SMSP13	SP
SMSU02	SU	SMAU08	AU	SMSP14	SP
SMSU03	SU	SMAU09	AU	SMSP15	SP
SMSU04	SU	SMAU10	AU	SMSP16	SP
SMSU05	SU	SMAU11	AU	SMSP17	SP
SMSU06	SU	SMAU12	AU	SMSP18	SP

Table S2. Climate data for the day of collection for each sample.

Sample	T (℃)	Po (mmHg)	U (%)	R (mm)
SMSU01	23.0	752.9	48.1	0
SMSU02	22.8	751.9	56.7	0
SMSU03	22.8	751.9	56.7	0
SMSU04	24.4	751.0	32.3	0
SMSU05	26.4	749.2	64.4	0
SMSU06	26.4	749.2	64.4	0
SMAU07	21.1	753.5	93.1	0
SMAU08	21.0	754.2	78.6	0
SMAU09	12.7	761.8	56.3	0
SMAU10	18.8	759.2	98.0	16.2
SMAU11	24.8	757.5	82.0	0.3
SMAU12	15.7	764.3	65.0	0
SMSP13	16.3	759.7	60.4	0
SMSP14	15.7	759.3	53.6	0
SMSP15	12.6	763.5	51.4	0
SMSP16	13.5	762.5	45.0	0
SMSP17	14.3	758.3	75.0	12.8
SMSP18	13.5	760.1	52.9	0

Table S3. Families with significant differences among groups

SP-SU	SP-AU	SU-AU
Chitinophagaceae	Chitinophagaceae	Rhodanobacteraceae
Enterobacteriaceae	Rhodanobacteraceae	Muribaculaceae
Rhodanobacteraceae	Ruminococcaceae	Flavobacteriaceae
Ruminococcaceae	Rhizobiaceae	Alcaligenaceae
Rhizobiaceae	Xanthomonadaceae	Gitt-GS-136
Lachnospiraceae	Lachnospiraceae	TK10
Mycobacteriaceae	Comamonadaceae	
Bacillaceae	Mycobacteriaceae	
Xanthobacteraceae	Bacillaceae	
Streptomycetaceae	Xanthobacteraceae	
Bifidobacteriaceae	Streptomycetaceae	
Helicobacteraceae	Helicobacteraceae	
Vagococcaceae	Beijerinckiaceae	
Beijerinckiaceae	Lactobacillaceae	
Oscillospiraceae	Micrococcaceae	
Erysipelotrichaceae	Nocardioidaceae	
Micrococcaceae	Halomonadaceae	
Nocardioidaceae	Microbacteriaceae	
Halomonadaceae	Woeseiaceae	
Woeseiaceae	Dietziaceae	
Hafniaceae	Kiloniellaceae	
Dietziaceae	Eubacterium_coprostanoligenes_group	
Kiloniellaceae	Unknown_Family	
Eubacterium_coprostanoligenes_group	Streptosporangiaceae	
Streptosporangiaceae	Flavobacteriaceae	
Gemmatimonadaceae	Geodermatophilaceae	
Flavobacteriaceae	Nocardiaceae	
Nitrosomonadaceae	67-14	
Geodermatophilaceae	Vibrionaceae	
Nocardiaceae	Polyangiaceae	
67-14	Thermomonosporaceae	
Vibrionaceae	Sutterellaceae	
Thermomonosporaceae	Labraceae	
Brevibacteriaceae	IMCC26256	
Labraceae	Akkermansiaceae	
Methyloligellaceae	Alicyclobacillaceae	
IMCC26256	Chloroplast	
KD4-96	Propionibacteriaceae	
Akkermansiaceae	Monoglobaceae	
Alicyclobacillaceae	Tsukamurellaceae	

Monoglobaceae	Alcaligenaceae
Tsukamurellaceae	Sandaracinaceae
Alcaligenaceae	RF39
Sandaracinaceae	Hungateiclostridiaceae
S085	Thermaceae
RF39	Acetobacteraceae
MB-A2-108	Frankiales
Hungateiclostridiaceae	EPR3968-O8a-Bc78
Caulobacteraceae	11-24
bacteriap25	Nakamurellaceae
Acetobacteraceae	Ferrovibrionales
TK10	Pectobacteriaceae
Frankiales	
EPR3968-O8a-Bc78	
11-24	
Nakamurellaceae	

Table S4. Spearman's rank correlation coefficient between gut microbiota and climatic factors (* $P < 0.05$, ** $P < 0.01$)

	Differential phyla	T	Po	U	R
SP-AU	Bacteroidota	0.060	-0.021	0.329	-0.009
	Chloroflexi	-0.530	0.102	-0.600*	-0.396
	Cyanobacteria	-0.557	0.464	-0.783**	-0.600*
	Gemmatimonadota	-0.320	0.194	-0.631*	-0.529
	Deinococcota	0.015	-0.150	0.225	0.104
SP-SU	Firmicutes	-0.457	0.540	0.084	0.393
	Bacteroidota	0.573	-0.611*	0.056	-0.393
	Acidobacteriota	-0.671*	0.713**	-0.673*	-0.356
	Chloroflexi	-0.803**	0.741**	-0.452	-0.044
	Gemmatimonadota	-0.543	0.538	-0.429	-0.317
	Verrucomicrobiota	0.259	-0.181	-0.620*	-0.481
	Patescibacteria	-0.579*	0.619*	0.004	0.420
SU-AU	Desulfobacterota	0.137	-0.263	-0.642*	-0.382
